# Prevalence and patterns of laxative use in subjects with self-reported constipation: results from a multinational digestive health survey

**DOI:** 10.1177/17562848241232605

**Published:** 2024-03-04

**Authors:** Brian E. Lacy, Régis Delfini, Bernward Fladung, Robert Lange

**Affiliations:** Division of Gastroenterology, Mayo Clinic, Jacksonville, FL, USA; Sanofi, Frankfurt, Germany; Freelance Medical Advisor, Linnich, Germany; Régis Delfini is currently affiliated to Boehringer Ingelheim, Ingelheim, Germany; Industriepark Hoechst, Building K 607, 65929 Frankfurt am Main, Germany

**Keywords:** constipation, digital survey, QoL, self-treatment, treatment satisfaction

## Abstract

**Background:**

Constipation is characterized by symptoms of straining, hard stool, difficult evacuation, and infrequent bowel movements. Online surveys provide valuable information about patients’ perspectives, symptoms, management, treatment satisfaction, and risk factors.

**Methods:**

This survey explored subject experiences involving 20 gastrointestinal (GI) conditions. In total, 20,099 respondents in seven countries with varied cultural and socioeconomic backgrounds participated. *Post hoc* analysis of ‘self-reported constipation’ and related symptoms experienced within the past 6 months and the last episode of constipation provided data on prevalence, demographics, frequency and duration of episodes and related symptoms, impact on quality of life (QoL), management with or without laxatives, and resulting treatment satisfaction.

**Results:**

In total, 10,425 subjects reported constipation within 6 months and 2637 at the last episode. Prevalence was highest in females and younger adults. Most subjects reported various coexisting GI symptoms. Almost 80% of 6865 episodes reported by 5337 subjects occurred every 2–3 months to every 2–3 weeks. A higher frequency of constipation correlated with a greater impact on QoL. On a 10-point scale, the mean impact was 6.4. More than 90% of respondents had episodes ranging from 1 day to 1 week. More than 90% took action; 16% used laxatives, of whom 80.3% were satisfied.

**Conclusion:**

Constipation, a highly prevalent disorder, spans cultures and socioeconomic classes. Its chronic recurrence has a significant impact on QoL, resulting in symptom self-management in >90% of subjects. Significantly higher satisfaction rates in subjects treated with than without laxatives reflect subjects’ reports that self-reported constipation can be treated effectively with laxatives.

## Introduction

Constipation symptoms vary from patient to patient but commonly include straining at stool, infrequent bowel movements, difficult evacuation of stool, and feelings of incomplete evacuation.^1^ Chronic constipation is common worldwide, with an estimated prevalence of 15%.^2^ The prevalence of self-reported constipation is generally higher than studies using the more stringent Rome criteria.^3,4^ In a recent large, multinational, internet- and population-based survey, more than 40% of persons worldwide reported functional gastrointestinal (GI) disorders. That survey, which used the Rome IV criteria involving 54,127 respondents, identified an 11.7% [95% confidence interval (CI): 11.4–12.0] prevalence of chronic (functional) constipation.^5^

Although the Rome criteria are considered the gold standard for diagnosing chronic constipation, symptoms of constipation and other associated symptoms are often perceived differently by subjects in the community.^6^ Beyond the criteria used to establish the definition of constipation, prevalence rates are also influenced by age, gender, socioeconomic factors, medications, comorbid conditions, and cultural factors.^1,7^ Affected subjects typically self-manage symptoms of constipation, instituting dietary changes and using over-the-counter (OTC) agents.^3,8^ The extent to which this occurs globally, however, is not well understood.

A variety of agents and remedies are readily available without a prescription, including fiber, stool softeners, laxatives, and non-pharmacological interventions. These products are typically used for self-management without guidance by healthcare professionals (HCPs).^3,9^ The perceived success of treatment varies from subject to subject and is influenced by age, gender, and patient perceptions of constipation severity and impact on life activities.^10^ A large survey demonstrated that 28% of participants were dissatisfied with treatment.^11^ Interestingly, OTC laxatives such as bisacodyl had even more favorable outcomes (in terms of the number needed to treat) than prescription medications.^12^

Given the frequency of use of OTC agents, it is important for clinicians to understand response rates and satisfaction with OTC agents to effectively treat patients. Here we report the results of a *post hoc* analysis of constipation-specific data involving 10,425 subjects with self-reported constipation within the past 6 months and 2637 subjects who reported constipation at the last episode, reflecting a current, clinically relevant, real-world situation. Compared to a previous survey conducted in seven countries evaluating the prevalence of constipation and laxative use in the treatment of self-defined constipation, the data of this survey were gathered prospectively in seven globally more representative countries, providing insights into self-management with a stronger emphasis on concomitantly reported associated symptoms.^4^ We also focused on the prevalence of constipation, the frequency and burden of symptoms, reported management strategies and treatments, and respective treatment satisfaction with an emphasis on laxative treatment *versus* treatment without laxatives.

## Methods

The Digestive Health Segmentation study was a global market research study conducted by an international market research company (ISM Global Dynamics, Germany, sponsored by Sanofi). This survey covered a broad spectrum of upper and lower digestive health symptoms and conditions. Data on participants’ views were captured *via* computer-assisted web interviews between March and April 2018 in seven countries. Surveyed countries included Italy (*N* = 3127 respondents), the United States (U.S.) (*N* = 3101), Russia (*N* = 3062), Germany (*N* = 3053), Japan (*N* = 2948), Mexico (*N* = 2883), and Vietnam (*N* = 2313). A representative screening process comprising 34,130 respondents aged 18 years and older with specific quotas was applied regarding age, gender, region, and employment status, to obtain a representative sample from each country. Additional quotas were employed, although specific data could not be obtained in all countries (e.g. income in Russia or ethnicity in the U.S.). The survey included questions related to (i) the presence or absence of symptoms and concern for any of 20 pre-coded distinctive digestive health symptoms/conditions of the upper and lower GI tract including hepatic and pancreatic conditions; (ii) taking any action related to digestive health symptoms or conditions (e.g. medication, home remedies, resting) in the past 6 months; (iii) openness to treatment with medication; and (iv) responsibility for purchasing digestive health products. The main questionnaire covered different topics: (i) demographics, lifestyle, and attitudes; (ii) digestive health suffering and concerns; (iii) last occasion recall; (iv) brand usage and awareness. The statistical analysis was based on subjects (*n* = 20,099) who reported symptoms and/or who took action to treat their symptoms. Subjects who reported symptoms of constipation were compared to those without constipation across a variety of parameters. The first part of the data analysis focused on demographics and prevalence data of subjects with (*N* = 10,425) or without (*N* = 9674) self-reported constipation within the past 6 months, including gender, age, and socioeconomic parameters (work status, education, income).

Data were analyzed in total and for subgroups (e.g. country, gender, and age groups). Analysis (total and subgroups) provided results on the assessment of self-reported constipation (including associated symptoms and symptom combinations) and quality of life (QoL). The frequency of constipation occurrence with impact on the daily life of sufferers with constipation alone compared to those with constipation plus accompanying symptoms was assessed. For the reason of simplification, better comprehension, and to facilitate the interpretation of differences, the three lowest frequency categories were summarized as ‘low frequency’, the highest three as ‘high frequency’, and the three categories in between as ‘mid-frequency’. Similarly, the impact on QoL categories was grouped into ‘low/no’ for the lowest four categories, ‘high’ for the three highest categories, and ‘mid’ for the three categories in between.

Respondents also provided information on their last episode of GI symptoms. The last episode recalls were used to investigate the frequency of concomitant symptoms and symptom combinations and the management of constipation and related symptoms, that is, taking prescription (Rx) or OTC medications including laxatives (stimulant or non-stimulant), supplements, medical devices, foods, vitamins, etc. and respective treatment satisfaction. A subgroup analysis was conducted by segregating respondents with constipation into three treatment subgroups (laxative users, those who took other actions but did not take laxatives, and those who did nothing) and two symptom subgroups (with and without recalled concomitant symptoms in association with constipation). In a further step, satisfaction with treatment in subjects who were treated with laxatives was compared to the majority of subjects who took actions (e.g. diet and lifestyle changes, medications) but not with laxatives. Both groups were also segregated into subgroups of subjects with constipation alone *versus* subjects who reported combinations with one or more other symptom(s).

## Results

### Part 1 – Demographics and prevalence data

**Figure 1.**
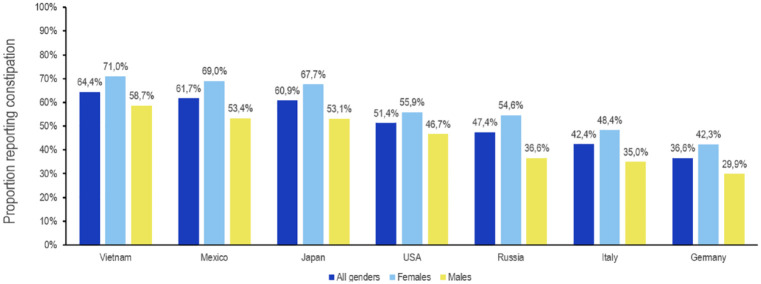
Prevalence (%) of self-reported constipation per country (total and by gender).

Of 20,099 individual respondents who completed the survey, 10,425 subjects reported symptoms of constipation (51.9%), while 9674 did not (48.1%). Responders were analyzed and compared for gender, age, work status, education, and income (Table 1). Overall (all seven countries combined), the prevalence of self-reported constipation was high in all countries although some differences were noted. The prevalence was highest in Vietnam, followed by Mexico, Japan, the U.S., Russia, Italy, and Germany (Figure 1). The proportion of females among the group of respondents reporting constipation was significantly higher than that of females not reporting constipation [60% *versus* 47% (*p* < 0.001)], whereas the proportion of males was significantly lower [40% *versus* 53% (*p* < 0.001)] (Table 1). Although the range between countries was considerable, with Russia (69%) and Italy (63%) having the highest, and Vietnam (51%) and the U.S. (56%) having the lowest proportion of females, the difference was statistically significant in all countries. Overall, and for most countries, a higher frequency of females with constipation was found for all age groups (Table 1). There were also other small but significant differences with regard to age (higher frequency of self-reported constipation in the lower age groups) and education (higher frequency in the higher education subgroup). On a country level, these differences were not found consistently.

**Table 1.** Demographics and prevalence data.

Characteristics	Total respondents (*N* = 20,099)		Respondents with self-reported constipation (*N* = 10,425)			Respondents without constipation (*N* = 9674)		
	Number	%	Number	%	S S^a^	Number	%	S S^a^
Gender								
Female	10,750	53	6227	60	w/o (<0.001)	4523	47	
Male	9349	47	4198	40		5151	53	w (<0.001)
Age (years)								
<29	4360	22	2488	24	w/o (<0.001)	1872	19	
30–44	6586	33	3645	35	w/o (<0.001)	2941	30	
45–59	5581	28	2673	26		2908	30	w (<0.001)
>60	3572	18	1619	16		1953	20	w (<0.001)
Work status								
Yes	13,639	68	7232	69	w/o (0.001)	6407	66	
No	6460	32	3193	31		3267	34	w (0.001)
Education								
Low	3010	15	1453	14		1557	16	w (0.026)
High	17,050	85	8953	86	w/o (0.027)	8097	84	
Dk/other	39	0	19	0		20	0	
Income (excl. MX)								
Low	5618	28	2849	27		2769	29	
Medium	7177	36	3606	35		3571	37	
High	3188	16%	1625	16%		1563	16	
Dk/other	1250	6%	547	5%		703	7	w (0.002)

a SS, statistical significance; Statistically significant differences were marked at the higher value, that is, either for ‘with’ *versus* ‘without’ (w/o) or for ‘without’ *versus* ‘with’ (w) constipation.

### Part 2 – Six-month symptom data

#### Frequency of constipation and related symptoms

Of 10,425 subjects who reported constipation in the past 6 months, more than three of four subjects suffered from a wide range of GI symptoms in addition to those of constipation (Table 2). The average number of symptoms experienced in the past 6 months was 10.0 for respondents with self-reported constipation *versus* 8.0 for total respondents and 5.6 for respondents not reporting constipation. Table 2 shows that most subjects with constipation also experienced other GI symptoms in association with constipation. More than 50% of subjects reported gas/flatulence/belching (76.9%), bloating/distension (65.4%), irregular bowel movements (62.3%), hard, dry stools (60.7%), dyspeptic symptoms, including nausea/upset stomach (59.8%), indigestion 58.6%, and belly cramps or belly pain below the umbilicus but not menstrual cramps (56.4%). These concomitant symptoms were consistently reported by subjects from all seven countries.

**Table 2.** Subjects with constipation experienced in the past 6 months (total and by gender), sorted by combination frequency.

Constipation and related symptoms	Total		Females (F)			Males (M)		
	Number	%	Number	%	S S^a^	Number	%	S S^a^
Constipation total	10,425	100	6227	100		4198	100	
Constipation and gas/flatulence/belching	8017	77	4844	78	M(0.018)	3173	76	
Constipation and bloating/distension	6816	65	4306	69	M(<0.001)	2510	60	
Constipation and irregular bowel movements	6490	62	4018	65	M(<0.001)	2472	59	
Constipation and hard, dry stools	6328	61	3771	61		2557	61	
Constipation and nausea/upset stomach	6235	60	3808	61	M(0.002)	2427	58	
Constipation and indigestion	6108	59	3533	57		2575	61	F(<0.001)
Constipation and belly cramps or pain below the belly button (not menstrual cramps)	5884	56	3628	58	M(<0.001)	2256	54	
Constipation and sustained pressure/pain	3753	36	2126	34		1627	39	F(<0.001)

a SS, statistical significance; Statistically significant differences were marked at the higher value, that is, either for ‘Females’ *versus* ‘Males’ (M) or for ‘Males’ *versus* ‘Females’ (F).

Overall, more females than males reported these additional potentially related symptoms in addition to constipation: gas/flatulence/belching (*p* = 0.02), bloating/distension (*p* < 0.001), irregular bowel movements (*p* < 0.001), nausea/upset stomach (*p* < 0.001), and belly cramps or belly pain below the umbilicus but not menstrual cramps (*p* < 0.001). A male predominance was recorded for constipation and indigestion (*p* < 0.001), and sustained pressure/pain (*p* < 0.001). On a country level, the gender distribution varied. Whereas in Russia, the gender distribution was balanced, in Japan, most of the symptoms displayed in Table 2 were reported more frequently by men than women, that is, constipation and hard, dry stools (67% *versus* 62%), indigestion (60% *versus* 55%), nausea/upset stomach (60% *versus* 55%), belly cramps or belly pain (61% *versus* 56%), and sustained pressure/pain (50% *versus* 34%). The analysis of additional symptoms reported, and age groups also found that more subjects of younger, compared to older, age groups reported concomitant, potentially related, symptoms in addition to constipation. This observation was consistent in all countries except Japan, where a predominance was seen in the age group 30–44 years (compared to age 29 and younger).

**Figure 2.**
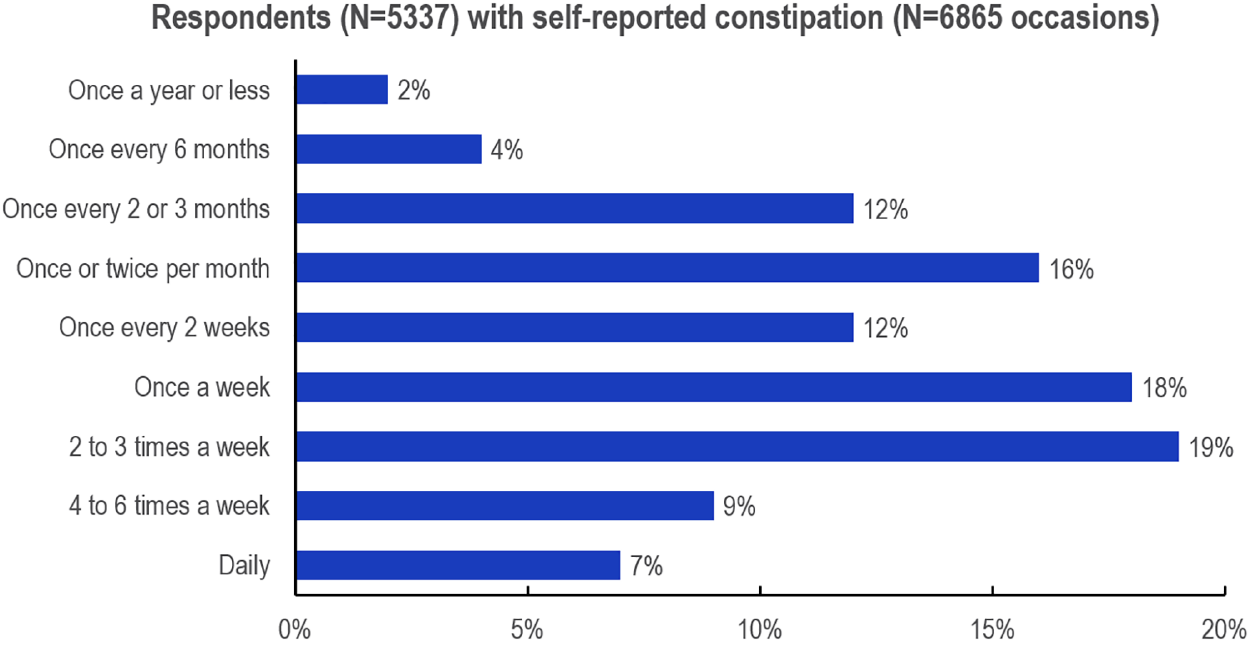
Frequency of constipation (%).

The distribution of frequency of self-reported constipation was very heterogeneous (Figure 2). Data were available from 5337 subjects and 6865 episodes of constipation. The frequency of constipation ranged from once per year or less to daily occurrence. The medium frequencies (two to three times a week to once every 2 or 3 months) were most often reported.

#### Quality of life

**Figure 3.**
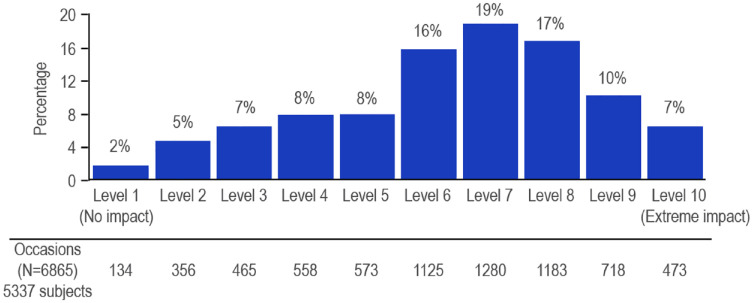
Impact of constipation episodes on QoL. QoL, quality of life.

An analysis of the impact of constipation determined that the majority of episodes had a medium to high negative impact on life. On a 10-point scale (with 0 = no impact and 10 = extreme impact), subjects grouped events most frequently into impact levels 6–9 with a mean impact on QoL of 6.4 per episode (Figure 3). Concerning gender and age, a slight tendency for females (mean impact 6.5) reporting higher impacts than males (6.1) and the age group of 60 and above (5.8) reporting less severe impact on life, than lower age groups, (6.3–6.7) was observed.

**Figure 4.**
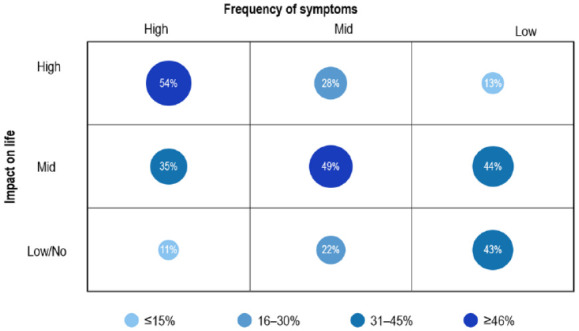
Correlation (%) of frequency groups and impact on QoL groups. QoL, quality of life.

The frequency of self-reported constipation was found to positively correlate with the impact on QoL. A significant correlation was observed, that is, the greater the frequency of constipation episodes, the more impact this condition had on individual life (5.0, 6.2, and 7.3 for low, mid, and high frequency, respectively). A similar association was also observed in the group of subjects who reported only constipation and no additional symptoms in the past 6 months with a total of 2846 observations. As displayed in Figure 4, a high frequency of constipation was associated with an increased impact on QoL. Similarly, a high impact on QoL was correlated with reduced stool frequency.

### Part 3 – Last episode data

#### Frequency of constipation and related symptoms

Of 19,327 last occasion recalls, 2637 subjects (13.6%) reported constipation either alone or in combination with other GI symptoms. Table 3 shows the frequency of additionally reported associated GI symptoms. Five symptoms had a frequency of >10% (hard, dry stools, gas/flatulence/belching, irregular bowel movements, bloating/distension, and indigestion). Subjects with constipation reported slightly more additional GI symptoms than those without: the average number was 2.1 for respondents with self-reported constipation *versus* 1.9 for total respondents and 1.6 for respondents not having reported constipation. The difference between means was statistically significant (*p* < 0.001).

**Table 3.** Subjects with constipation experienced in the last episode (total and by age) sorted by combination frequency.

Constipation and related symptoms			Respondents with self-reported constipation											
	Total		<29 years (a)			30–44 years (b)			45–59 years (c)			>60 years (d)		
	No.	%	No.	%	S S^a^	No.	%	S S^a^	No.	%	S S^a^	No.	%	S S^a^
Constipation total	2637	100	554	100		889	100		748	100		446	100	
Constipation, individual symptom	884	34	132	24		272	31	a(0.004)	268	36	a(<0.001) b(0.032)	212	48	all(<0.001)
Constipation combination symptom	1753	66	422	76	b(0.004) c,d(<0.001)	617	69	c(0.032) d(<0.001)	480	64	d(<0.001)	234	52	
Constipation and hard, dry stools	685	26	145	26		228	26		205	27		107	24	
Constipation and gas/flatulence/belching	602	23	156	28	b(0.033) d(<0.001)	205	23	d(0.001)	176	24	d(<0.001)	65	15	
Constipation and irregular bowel movements	526	20	107	19		166	19		171	23		82	18	
Constipation and bloating/distension	507	19	117	21	d(0.015)	183	21	d(0.008)	139	19		68	15	
Constipation and indigestion	313	12	95	17	b(0.036) c(0.002) d(<0.001)	113	13	d(<0.001)	79	11	d(0.004)	26	6	
Constipation and belly cramps or pain below the belly button (not menstrual cramps)	218	8	58	10	d(0.022)	68	8		67	9		25	6	
Constipation and nausea/upset stomach	212	8	64	12	c(0.016) d(<0.001)	79	9	d(<0.001)	58	8	d(<0.001)	11	2	
Constipation and sustained pressure/pain	96	4	14	3		41	5	d(0.008)	32	4		9	2	

a SS, statistical significance; Statistically significant differences between age groups were marked at the higher value as applicable, that is, either *versus* ‘<29 years’ (a) or *versus* ‘30–44 years’ (b) or *versus* ‘45–59 years’ (c) or *versus* ‘>60 years’, (d) or *versus* all other age groups (all).

The highest proportion of respondents with concomitant symptoms at their last GI episode was found in Germany (73%), followed by the U.S. and Russia (68%), Vietnam (66%), Japan and Mexico (64%), and Italy (63%). Overall, the proportion between males and females experiencing concomitant symptoms was balanced without statistically significant differences in any country, with a slight predominance of females reporting bloating/distension [22% *versus* 15% (*p* < 0.001)] and gas/flatulence/belching (25% *versus* 20%, *p* < 0.001).

Subjects belonging to younger age groups consistently reported more additional symptoms than older study participants (see Table 3). Although there were differences between individual countries concerning the frequency and distribution of additional individual symptoms, the observation of younger people more frequently reporting additional GI symptoms was made in all countries.

#### Treatment satisfaction

**Figure 5.**
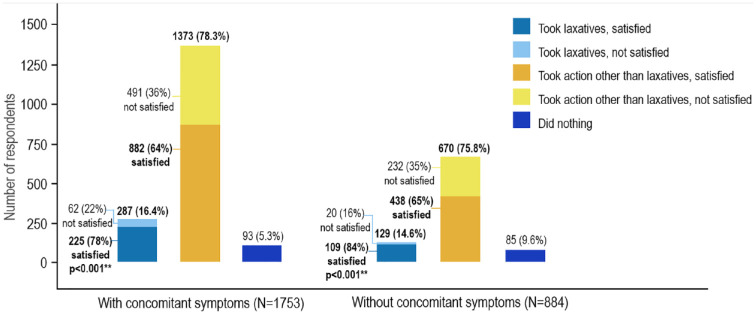
Overview of the treatment mode and treatment satisfaction* in subjects with self-reported constipation with and without further symptoms during the last episode of GI complaints. *Respondents had the choice between five options: (1) not satisfied at all, (2) not very satisfied, (3) neutral, (4) somewhat satisfied, and (5) very satisfied. To ease the interpretability of results, options 1–3 were summarized as ‘not satisfied’ and options 4–5 as ‘satisfied’. **Statistical difference in the proportion of satisfaction with treatment: laxative users *versus* group taking action other than laxatives.

When questioned about the chosen treatment, more than 90% of 2637 subjects with constipation during their last episode took action but only a minority took laxatives (see Figure 5). The most frequently mentioned action among those who did not take laxatives was consuming fiber-rich foods (31%) or probiotic-enriched foods (28%), taking a probiotic supplement (25%), using a prescription medication (18%) or using a medication not prescribed by a doctor (20%). A higher proportion of respondents without further concomitant symptoms did not use any treatment compared to those with additional symptoms (10% *versus* 5%, *p* < 0.001).

A higher proportion of subjects who were treated with laxatives were satisfied with treatment compared to those who took actions, but not with laxatives. Of 416 subjects who took laxatives at onset, 334 (80.3%) were satisfied and 82 (19.7%) were not satisfied. In comparison, of 2043 subjects who took any action, but did not take laxatives, 1320 (64.6%) were satisfied and 723 (35.4%) were not satisfied. The differences were also statistically significant in the subgroups with (78% *versus* 64%, *p* < 0.001) and without (84% *versus* 65%, *p* < 0.001) concomitant symptoms. In the group of subjects who were self-treated with laxatives, the subgroup without concomitant symptoms reported more satisfaction with treatment (84%) than those with concomitant symptoms (78%), although this was not statistically significant. Results concerning satisfaction with treatment with laxatives or other actions (stratified according to subjects with and without concomitant symptoms) are displayed in Figure 5.

#### Duration of symptoms

The duration of the self-reported last episode of constipation among those who took actions, either self-treated with laxatives or not, was generally short. The duration was between 1 and 7 days in approximately 90% of subjects, while 10% of subjects or less had a duration of more than 1 week, whether they had additional symptoms or not (Table 4).

**Table 4.** Overview of duration of self-reported constipation in the last episode of GI complaints.

	Respondents with self-reported constipation in the last episode					
	Treated with laxatives (at onset)					
	With concomitant symptoms (*N* = 287)			Without concomitant symptoms (*N* = q129)		
	No.	%	S S^a^	No.	%	S S^a^
Duration						
1 day or less	141	49	w/o (0.00)	35	27	
>1 day but ⩽1 week	118	41		87	67	w (0.000)
>1 week but ⩽1 month	8	3		2	2	
>1 month	20	7		5	4	

a SS, statistical significance; Statistically significant differences were marked at the higher value, that is, either for ‘with’ *versus* ‘without’ (w/o) or for ‘without’ *versus* ‘with’ (w) constipation.

## Discussion

This population-based multinational survey investigated 20 distinct GI conditions and symptoms experienced in the 6 months prior to the survey in addition to the last episode recalled. Our analysis focused on the multinational participant perspective of constipation self-reported by respondents of highly different cultural backgrounds, socioeconomic status, and a wide spectrum of demographic variables. The objective was to obtain new insights into participants’ perception of constipation and its management because these patient-reported outcomes better reflect the real-world situation as opposed to the artificial confines of a clinical trial. As this survey addressed a multitude of upper and lower GI conditions and not just constipation, we believe that the data represent an accurate portrayal of the average patient with constipation.

The study reported here comprised 20,099 evaluable respondents, of whom 10,425 (51.9%) reported that they had symptoms of constipation within the past 6 months, while 2637 (13.1%) respondents experienced constipation either alone or combined with other symptoms during the last recalled GI complaint episode. A previous publication from 2008 presented the results of a comparable but considerably smaller market research study. That study comprised 13,879 adults from seven countries (U.S., UK, Germany, France, Italy, Brazil, and South Korea) of whom 1712 subjects (12.3%) experienced constipation in the past 12 months.^4^ Unlike that study the current project included two countries from Asia, and also Mexico and Russia, and thus our results represent a more balanced global perspective, less dominated by European countries. These two factors strengthen the value of this data. Furthermore, subjects could report episodes of constipation without accounting for other constipation-specific symptoms as is common for occasional constipation.^13^

### Demographics

Female gender, advanced age, lower socioeconomic status, lower parental education rates, stress, and lack of physical exercise are the most frequently cited demographic risk factors associated with chronic constipation.^14,15^ The prevalence of self-reported constipation observed in this survey was high in all countries but varied strongly between 36.6% (Germany) and 64.4% (Vietnam). The predominance of female gender was confirmed in this survey across all countries (ranging from 51% to 69%). However, this survey identified a higher prevalence of self-reported constipation in younger adult age groups. This finding may be related to the reported different comparative epidemiology of constipation subtypes and could reflect a high prevalence of occasional constipation which is the most common self-diagnosed and self-reported condition, not restricted to stool frequency or symptoms defined by Rome criteria.^13^ Compared to chronic constipation, it is more frequently found in younger adults, whereas chronic constipation and constipation secondary to organic cause or medication use have a higher preponderance in older subjects.^16–18^ The prevalence of functional constipation (FC) decreases with advancing age, as shown in a study conducted on more than 10,000 U.S. adults^19^ and confirmed by the large, multinational, internet- and population-based survey recently published by Sperber *et al.*^5^ As self-reported constipation was not specified further, the proportion of different subtypes was not controlled for in our study but may likely have been dominated by ‘FC’. The prevalence of constipation was also higher in younger (<40 years) and older (⩾70 years) age groups compared to medium age groups (⩾40 up to 70 years) in an epidemiological study comprising 15,002 individuals in Germany.^20^ Younger age groups also reported concomitant GI symptoms more frequently, reflecting the symptom-based subjective nature of constipation as a medical condition. In contrast to other studies, no meaningful influence of socioeconomic factors (work status and income) and education level could be detected in the current extensive survey (Table 1). It is possible that our survey reflects subjects with a higher education level and an economically more secure position compared to the general population.

### Data on constipation in the past 6 months

Of the 10,425 subjects reporting constipation in the past 6 months, >75% also experienced one or more symptoms known to be associated with constipation in the same period.^7,21^ Specifically, those GI symptoms likely related to constipation have each been reported by more than 50% of constipated subjects, that is, gas/flatulence/belching, bloating/distension, irregular bowel movements, hard, dry stools, nausea/upset stomach, indigestion, and belly cramps or pain. The number of average additional GI symptoms was considerably higher in respondents with reported constipation (10.0) than those without (5.6), confirming the recognition of constipation as a poly-symptomatic disorder, including various aspects of disturbed defecation.^22^

Related symptoms such as bloating play a major role in irritable bowel syndrome (IBS)-constipation (IBS-C) and FC because of their association with painful constipation and abdominal pain, more so in IBS-C than in FC.^23,24^ Bothersome symptoms of constipation, including abdominal discomfort and bloating, may explain the strong impact on QoL with a mean impact of 6.4 to participants of this survey on a 0–10 scale. Similar results were reported by Wald^4^ using the SF-36 questionnaire.^4^

The frequency of self-reported combination and impact on life (QoL), both gathered in this survey, were analyzed to assess whether a higher frequency of constipation episodes was associated with a greater impact on life compared to a single or rare occurrence of these events. Figure 4 shows a significant correlation between both parameters, that is, the higher the frequency of constipation episodes, the greater the impact on QoL. It also shows that this association was strong in both directions. Adequate symptomatic treatment of constipation by reducing the frequency of occasions can therefore successfully improve QoL. This positive impact of laxatives on QoL may explain why laxatives are used off-label for the prevention of constipation, sometimes even on a daily basis.^3^

### Data on constipation at the last episode

Subjects who reported constipation as a symptom at their last recall episode, either alone or in combination with other related symptoms, were of special interest because the recollection of symptoms, actions taken, and other details are considered to be the most reliable compared to chronologically more distant episodes. This was not only shown for the recollection of pain symptoms as measured in analgesic studies^25^ but was also shown for constipation-related measurements.^26^ The majority of subjects who self-reported constipation at their last GI episode also experienced additional GI symptoms likely related to constipation. Overall, the survey results show that the combination of constipation with other GI symptoms is not a local but a universal phenomenon, ranging from 63% to 73% in all surveyed countries, confirming its nature as a poly-symptomatic disorder.^22^ These findings support the use of the Rome IV criteria, which are predominantly symptom based, as patients with constipation not only have infrequent stools but also other symptoms including straining at stool, a feeling of incomplete evacuation, a need for digital assistance to evacuate stool, bloating, and hard or lumpy stools.^2,27^

As the data on the co-occurrence of single concomitant symptoms reflected only one episode, additional symptoms were reported less frequent than for the respective 6-month period (see Table 3). Subjects with self-reported constipation reported more additional GI symptoms (2.1) than those without constipation (1.6).

Little information is available regarding subjects’ self-management of constipation. This multi-national survey identified a host of actions or treatments, including both pharmacological (prescribed or OTC laxatives, fiber, probiotic supplements, alternative medicines like herbal remedies) and non-pharmacological interventions (e.g. dietary and lifestyle changes or acupuncture). More than 90% of subjects with constipation at the last episode reported having taken action, though only 16% used laxatives to treat their symptoms. This is similar to the Wald study, where only a minority of subjects reporting constipation symptoms reported laxative use.^4^ The vast majority of treaters were satisfied with the laxative treatment they took, regardless of whether they had experienced concomitant symptoms or not. The satisfaction with treatment was slightly higher in patients not reporting additional symptoms, indicating the beneficial effect of laxatives even in subjects suffering from multiple symptoms concomitant with constipation. This slight difference in satisfaction may also be related to the different frequency of concomitant symptoms in different age groups. Regardless of whether or not constipation was reported with concomitant symptoms, those respondents who were treated with laxatives were more satisfied with treatment than those who took actions, but not with laxatives. The differences were also statistically significant in the subgroups with and without concomitant symptoms.

Overall, this survey showed high satisfaction with treatment in the population that decided to treat (even higher with laxative treatment than with treatment without laxatives), which is consistent with data from an internet survey that showed relatively low dissatisfaction (17%) in women treating self-reported chronic constipation.^28^ Analysis of pooled data from two randomized, placebo-controlled trials found that bisacodyl and sodium picosulfate significantly improved not only bowel habit parameters but also QoL, in patients with chronic FC.^29^ High satisfaction among subjects treating constipation with laxatives reflects their focus on symptom relief which is appropriate for OTC medications and signals that the reported concomitant symptoms were genuinely linked to constipation in most cases. What may have contributed to satisfaction in our survey was the finding that the duration of the last episode with constipation (with or without additional symptoms) was only up to 1 week in more than 90% of subjects. However, several internet survey results,^11,28,30^ and a *post hoc* analysis using patient-reported data,^8^ showed that overall, satisfaction with laxatives and fiber products, either available as prescription or OTC, was limited. Fiber and bulk laxatives provided less, while stimulant laxatives provided more, satisfaction,^8,30^ probably due to the latter’s faster and reliable onset of action, as the reason for dissatisfaction appears mostly related to efficacy.^30^

Insufficient counseling by HCPs on OTC laxatives and often suboptimal management of constipation resulting in inappropriate use may also represent an underestimated reason for considerable patient dissatisfaction.^3,31^

## Conclusion

The data on self-reported constipation in the past 6 months and at the most recent episode demonstrate that most subjects also experience additional GI symptoms possibly related to constipation, confirming clinical experience, and some definitions, that recognize constipation as a poly-symptomatic disorder. Frequently reported associated symptoms included (some of which are part of the definition of constipation) hard, dry stools, gas/flatulence/belching, irregular bowel movements, bloating/distension, indigestion, nausea/upset stomach, and belly cramps or pain. The overall predominance of these symptoms was more or less observed across all countries, explaining the relevance of constipation for QoL impairment. Almost 80% reported a frequency of constipation episodes between once every 2–3 months and every 2–3 weeks, demonstrating that chronic recurrence of constipation is a major health issue for concerned subjects. This was highlighted by the negative impact on QoL with a higher frequency of constipation episodes emphasizing the need for treatment that reliably resolves or eases the symptoms of constipation.

Reducing the frequency and severity of constipation episodes should be the main objective in the management of constipation. Interestingly, while more than 90% of respondents with constipation at their last GI episode reported having taken action, only a minority treated their episode with laxatives, regardless of whether they had concomitant symptoms or not. Importantly, those who did were more satisfied with treatment compared to those who took any other action but did not treat themselves with laxatives, leading to the conclusion that treatment with laxatives effectively treats symptoms of constipation on a subjective level.

Like all survey studies, our study has several limitations worth mentioning. One, ‘constipation’ was not explicitly pre-defined, thereby allowing respondents to apply their criteria. The data reported here reflect a global, multicultural experience and is different from clinical trials using the more restrictive Rome IV criteria. Two, questions in the survey were not validated using a patient focus group. However, the questions were straightforward, were not complex, and have been used with success in other constipation survey studies. Three, similar to other surveys, this survey had selection bias toward individuals who have access to the internet, resulting in more young and middle-aged subjects with higher education levels and likely to be more physiologically healthy and mentally fit compared to the general population.^4,11^ Four, based on the significant cultural and socioeconomic differences between selected countries, this survey comprises heterogeneous populations reflecting a more global view than previous publications. Five, the potential impact of concomitantly taken medications on bowel frequency could not be established. However, an inherent lack of scientific guidance and specificity concerning the questions on GI signs and symptoms is associated with online survey where participants were questioned. This may have contributed to an unknown blurring effect potentially increased by the population differences between countries.

In summary, constipation is a highly prevalent condition comprising a number of related GI symptoms which according to the severity and frequency of episodes has a considerable impact on QoL. Treatment, rather with laxatives than without, appears to be an effective measure to manage constipation and to cope with its bothersome symptoms. Further research focusing on the effectiveness of different management strategies to increase QoL and to ensure treatment satisfaction is needed to better counsel patients with constipation regarding treatment and self-treatment of symptoms of constipation.

## Declarations

**Ethics approval and consent to participate:** Not applicable.

**Consent for publication:** Not applicable.

**Availability of data and materials:** Qualified researchers may request access to data and related study documents including the study report, study protocol with any amendments, statistical analysis plan, and dataset specifications. Further details on Sanofi’s data sharing criteria, eligible studies, and process for requesting access can be found at https://www.vivli.org/.
